# Unlocking motor reserve: behavioral and neuroimaging correlates of locomotor adaptability from youth to old age

**DOI:** 10.3389/fnsys.2025.1686509

**Published:** 2025-11-10

**Authors:** Stefanie Kögler, Shawn Hiew, Robert Peach, Magnus Schindehütte, Mirko Pham, Carine Nguemeni, Daniel Zeller

**Affiliations:** 1 Department of Neurology, University Hospital of Würzburg, Würzburg, Germany; 2 Department of Neuroradiology, University Hospital of Würzburg, Würzburg, Germany

**Keywords:** aging, motor reserve, motor adaptation, physical activity, brain reserve

## Abstract

**Background:**

Aging humans show a remarkable heterogeneity in agility, dexterity, and mobility. Once motor functions are impaired, their restoration may be challenging or even impossible. Thus, it is crucial to anticipate and address declines in mobility due to age- and disease-related reductions in cognitive and motor resources. Quantification of the individual motor adaptive capacity, or “motor reserve” (mR), is essential for the development of innovative treatments enhancing this reserve.

**Objective:**

To assess whether biographical, behavioral, and neuroimaging measures can serve as quantifiable indicators of mR.

**Methods:**

Twenty-four healthy adults aged 21–80 years underwent cross-sectional assessment. Adolescent and recent physical activity was gathered via questionnaires. Current adaptability was investigated using a locomotor adaptation task on a split-belt treadmill and a visuomotor adaptation hand task. Magnetic resonance imaging (MRI) was used to assess grey matter volume and resting-state functional connectivity in motor and locomotor brain centres.

**Results:**

As expected, six-minute walking distance declined with age, largely reflecting reductions in grey matter volume. In contrast, motor adaptation abilities were preserved across the age span. Locomotor adaptation rates were positively associated with structural and functional integrity of motor networks, which were, in turn, influenced by long-term physical activity.

**Conclusion:**

Variability in motor adaptability among older adults may be explained by individual differences in motor network integrity and lifelong physical activity. The findings of this exploratory cross-sectional study support the potential of MRI-based connectivity measures and locomotor adaptation performance as surrogate markers of motor reserve. Enhancing mR through targeted interventions may help sustain mobility and functional independence in aging populations.

## Introduction

Over the past century, rising life expectancy has led to a growing global population of older adults. This demographic shift is both a public health success and a challenge, as aging is accompanied by gradual declines in cognitive and motor function, increasing the risk of impairments such as cognitive decline, mobility loss, and falls (Li et al., 2021). These risks are further compounded by neurodegenerative diseases that accelerate the depletion of neural resources. Nevertheless, many older individuals retain the ability to adapt to physical, environmental, and neurological changes (Krakauer, 2009; Sato and Choi, 2022; Scherder et al., 2007). This ability, known as motor adaptation, is the ability to adjust motor patterns in response to such alterations and is crucial for preserving function and supporting healthy aging. Advancing our understanding of these adaptive mechanisms may enable the development of interventions that enhance intrinsic capacity in later life. To do so, it is crucial to establish normative tools to quantify motor reserve (mR) across the lifespan and in clinical populations.

Similar to cognitive reserve, motor reserve (mR) may represent a threshold for functional decline in the motor domain, shaped by both quantitative brain measures and lifetime motor experiences (Zeller et al., 2024; Barulli and Stern, 2013). In this view, mR reflects the cumulative structural and functional brain changes acquired over life, providing resilience against age- and disease-related declines in motor function (Zeller et al., 2024; Palermo et al., 2025).

Multiple approaches can be considered for estimating mR. Drawing from the concept of cognitive reserve (Barulli and Stern, 2013), a biographical approach assesses physical activity across life stages. While valuable for capturing lifelong influences, this method yields a static estimate and is unsuitable as an outcome measure for interventions.

In contrast, behavioral approaches quantify mR based on current adaptive capabilities. These approaches typically involve introducing a controlled perturbation necessitating an update of existing motor programs. In visuomotor adaptation paradigms, rotating the screen cursor during a virtual reaching task elicits compensatory adjustments (Krakauer et al., 2005; Nguemeni et al., 2021). In locomotor adaptation, walking on a split-belt treadmill, where belts move at different speeds, necessitates recalibration of gait patterns (Malone et al., 2012; Malone and Bastian, 2010; Reisman et al., 2005; Sato and Choi, 2022). In both cases, the perturbation induces motor errors that diminish over time, reflecting adaptation. When the perturbation is removed, the resulting error in the opposite direction reflects context-dependent after-effects, which also fade over time (Bastian, 2008; Sato and Choi, 2022). The magnitude of the error along with the rates of adaptation and deadaptation are considered behavioral indicators of individual adaptive capacity.

Motor adaptation relies on the flexible recruitment of neuronal networks via existing or newly formed connections, resulting in a more efficient integration of functional brain networks. The ability to adapt, and consequently the mR, is influenced by factors such as age (Bruijn et al., 2012), central nervous system (CNS) morphology (e.g., brain volume) (Cassady et al., 2018), and functional connectivity (Kim and Kang, 2018). Thus, neuroimaging presents a third approach to directly probe the neural substrates of mR.

This study aims to: (i) define a set of mR measures, including biographical, behavioral, and neuroimaging metrics; (ii) assess the relationship of these measures with age; and (iii) investigate their interrelation and potential complementarity as quantifiable descriptors of mR. To our knowledge, no prior study has systematically evaluated motor adaptive capacity in this integrated manner, linking neural, behavioral and lifestyle factors across the adult lifespan. The present findings will contribute to a comprehensive framework for understanding how mR is established, maintained, and potentially enhanced.

## Methods

### Participants

Twenty-four adults (age-range 21–80) were recruited for this study according to the following criteria: normal performances in the Montreal Cognitive Assessment [MoCA (Nasreddine et al., 2005), score ≥26], the Frontal Assessment Battery [FAB (Dubois et al., 2000), score ≥ 12], the Functional Gait Assessment [FGA (Wrisley and Kumar, 2010), score ≥ 23], Timed Up-and-Go [TUG (Podsiadlo and Richardson, 1991), time < 20 s], the Jebsen-Taylor hand function test [JT (Hackel et al., 1992; Jebsen et al., 1969), score < 2], and the ability to walk overground without apparent asymmetry. Exclusion criteria included any history of neurological or psychiatric disorders, use of centrally acting medications, pregnancy, or contraindications to magnetic resonance imaging (MRI).

The study conformed to the principles of the declaration of Helsinki and was approved by the local ethics committee of the Medical Faculty at the University of Würzburg (74/20-am). All methods were performed in accordance with the relevant guidelines and regulations. All participants gave their written informed consent before participating in the experiments.

### Questionnaires

Depressive symptoms were assessed using the Beck Depression Inventory (BDI; Beck et al., 1961), which ranges from 0–63, with scores <13 indicating no depression. The BDI is validated for adults aged 18–80 (Beck et al., 1961). Handedness was measured with a modified Edinburgh Handedness Inventory (Oldfield, 1971), yielding scores from −20 (left-handed) to 20 (right-handed); scores >13 indicate right-handedness, −13 to 13 ambidexterity, and <−13 left-handedness.

Cognitive reserve was evaluated using the Cognitive Reserve Index Questionnaire (CRIq) (Nucci et al., 2012). The number of years during which tasks were performed for the three sections of education, working activity and leisure time were recorded and converted into normative scores using the author’s calculation tool. This questionnaire has been validated and found reliable for adults aged 18–80 in several prior studies (Farran and Darwish, 2023; Ozakbas et al., 2021). Physical activity was measured using a modified version of the Historical physical activity questionnaire (HistPAQ) (Kriska et al., 1988). This tool records various sports and activities that were performed across different life stages (primary school; ages 14–21, 22–34, 35–50, 50+) and the amount of time spent on each. Estimation of the energy expenditure was calculated using the 2024 Compendium of Physical Activities (Herrmann et al., 2024).

Furthermore, the Simple Physical Activity Questionnaire (SIMPAQ) (Rosenbaum et al., 2016) was used, which quantifies activity during the week preceding the study by leading a structured interview assessing number of hours spent sleeping, sitting down, doing house work and performing physical activity. The two questionnaires have been validated and applied in several prior studies (Dubose et al., 2007; Winter-Hart et al., 2004; Kriska et al., 2006; Rosenbaum et al., 2020; Schilling et al., 2018).

### Clinical assessments

All participants underwent clinical assessment of cognitive and motor functions. Frontal lobe function was assessed through FAB and cognitive impairment was ruled out through MoCA. Hand function was assessed using the Jebsen-Taylor hand function test. Postural stability and balance during walking were evaluated through FGA and TUG and the 25-foot-walk-test (25-FWT) was timed to calculate a participant’s maximum walking speed. To assess overall neuromuscular and cardiovascular capacity, participants completed the 6-Minute Walk TEST (6MWT). During overground walking, spatiotemporal parameters were recorded using the G-Walk 2 wireless system of gait analysis and processed using the software program BTS G-Studio (BTS Bioengineering, Milanese, MI, Italy).

### Visuomotor adaptation task

**Figure 1:**
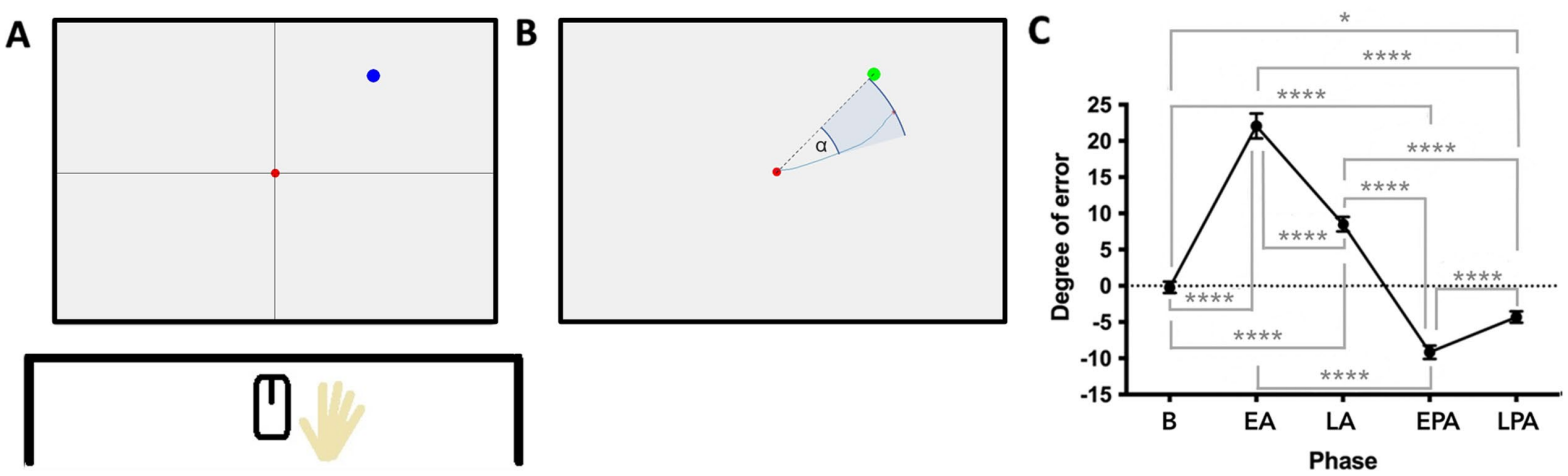
**(A)** Experimental setup of the VAT. Participants controlled a computer mouse with their preferred hand, which was hidden from view by a horizontal shield. A red dot appeared at the center of the screen, followed by a blue target dot in one of eight pseudo-randomized positions. Participants were instructed to first click the center dot and then move the cursor in a straight, continuous motion toward the blue dot. **(B)** When the cursor reached the blue dot—or any point at the same radial distance from the center—the target turned green, disappeared, and reappeared in a new location. The primary outcome measure was the angle of deviation (*α*), calculated at the midpoint between the center and the target. **(C)** Mean angular deviation (in degrees) across VAT phases. B, baseline; EA, early adaptation; EPA, early postadaptation; LA, late adaptation; LPA, late postadaptation. *p < 0.05; **p < 0.01; ***p < 0.005; ****p < 0.001.

The visuomotor adaptation task (VAT) was performed on a computer using a MATLAB R2017a programme. Participants were seated approximately 70 cm in front of a computer screen (47.5 cm width, 29.25 cm height) and controlled a computer mouse with their preferred hand which was occluded from view by a horizontal cardboard shield (Figure 1A). No mouse training was performed prior to the start of the task.

Each trial began with a red dot at the center of a white screen, followed by the appearance of a blue target dot in one of eight pseudo-randomized positions arranged in a circular layout. Participants were instructed to click the red center dot, before moving the cursor in a fluid movement at medium speed towards the blue dot without stopping or changing direction. Once the cursor crossed the invisible target circle’s radius, the blue dot turned green (regardless of cursor accuracy) and disappeared before reappearing in a new location.

The adaptation task consisted of three phases: baseline (3 blocks), adaptation (7 blocks), and washout (3 blocks). Each block comprised 4 cycles of the 8 target positions, totalling 32 trials. Blocks were separated 10 s and phases were separated by a minute, during which instructions were repeated.

During baseline and washout, cursor movement was displayed one-to-one with hand movement. During the adaptation phase, a 30° clockwise rotation was applied to the cursor trajectory relative to the red center point. The angular deviation error was measured as the angle between the straight line from the red center to the target and the cursor trajectory, recorded halfway between the center and the target (Figure 1B).

Key outcome measures included:

- Baseline: mean adaptation error during last block of baseline phase,
- Adaptation degree: difference between early and late adaptation errors (EA – LA),
- Deadaptation degree: difference between early and late post-adaptation errors (EPA – LPA),
- After-effects: difference between late adaptation and early post-adaptation errors (LA – EPA),
- Adaptation rate: adaptation degree divided by the number of adaptation trials,
- Deadaptation rate: deadaptation degree divided by the number of washout trials.

### Locomotor adaptation task

**Figure 2:**
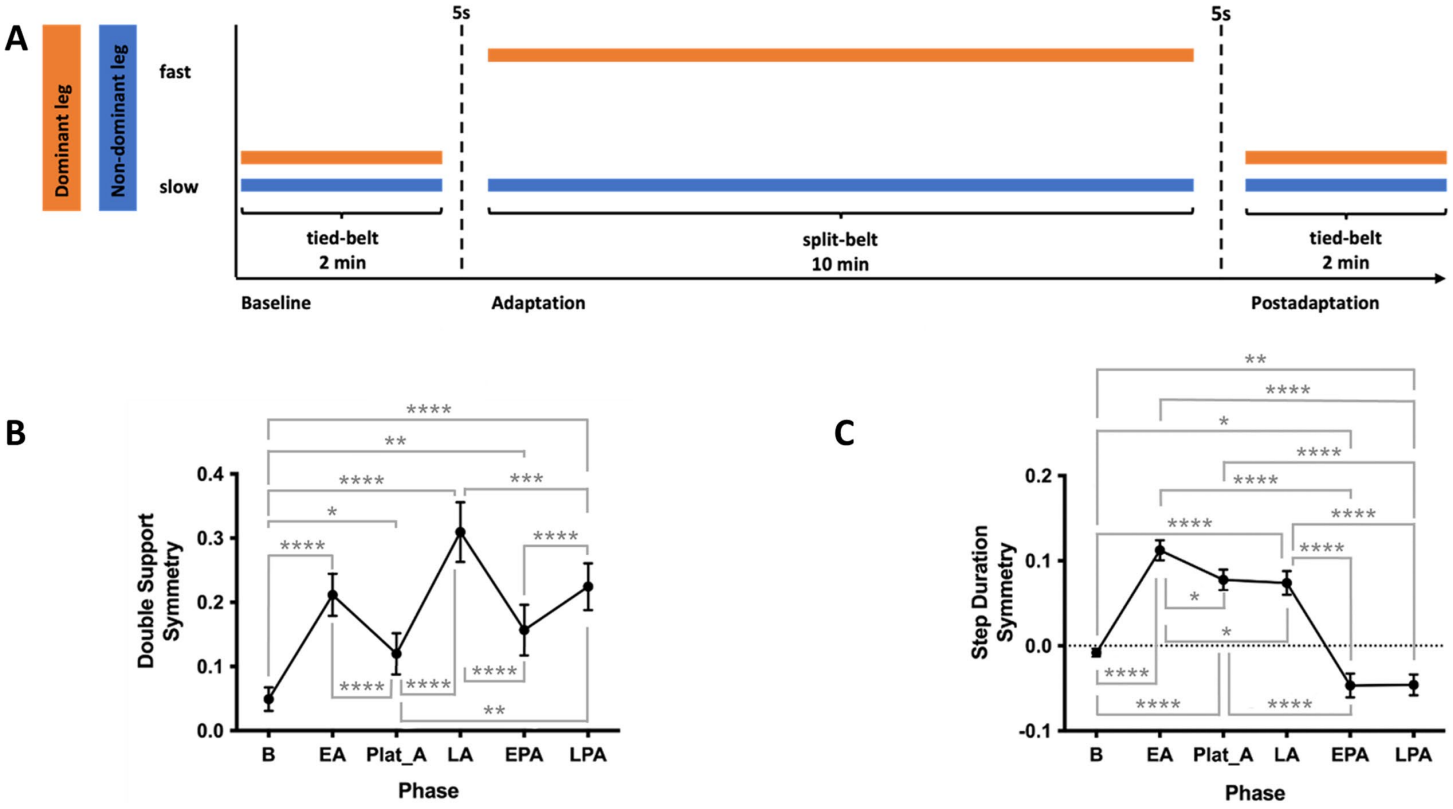
**(A)** Locomotor adaptation task (LAT) paradigm. Participants first walked for 2 min in a tied-belt condition at a slow speed (half their maximum speed as determined by the 25-Foot Walk Test, 25-FWT). This was followed by a 10-min split-belt adaptation phase, with the dominant leg on the fast belt (maximum 25-FWT speed) and the non-dominant leg on the slow belt. Finally, participants walked for 2 min in a postadaptation phase under the same slow tied-belt condition. **(B)** Double support (DS) symmetry across phases of the LAT **(C)** Step duration (SD) symmetry across phases of the LAT. SD, step duration. EA, early adaptation; EPA, early postadaptation; LA, late adaptation; LPA, late postadaptation; Plat_A, adaptation plateau. *p < 0.05; **p < 0.01; ***p < 0.005; ****p < 0.001.

The locomotor adaptation task (LAT) was performed on a custom split-belt treadmill (SBT; Woodway, Waukesha, WI, USA). The side of the fast belt was assigned based on foot preference: the foot used to kick a ball, or if undefined, the leg typically used to regain balance when falling forward. The fast speed was set to the participant’s maximum walking speed as determined by the 25-Foot Walk Test (25-FWT). The mean of two trials of the 25-FWT was taken where participants were instructed to walk at their maximum walking speed overground. Safety during the tests was ensured by ample space and a supervisor on the side, timing was recorded by hand. The slow-speed was set to half of that value (Figure 2A).

Participants wore comfortable clothing and shoes and were secured with a safety harness throughout the task. While walking, they held a horizontal handrail positioned in front of them, with instructions not to lean on it. They were also asked to maintain gaze at eye level on a fixed point on the wall and avoid looking down during the task.

Participants first walked for 2 min with both treadmill belts at the slow speed (baseline). This was followed by a 10-min adaptation phase, during which the belt under the dominant leg moved at the fast speed while the other remained slow. Finally, participants completed a 2-min post-adaptation phase with both belts tied at the slow speed. The experimental paradigm is illustrated in Figure 2A.

Spatiotemporal gait parameters were recorded at 100 Hz using Moticon OpenGo sensor insoles and analyzed using the associated desktop software (Moticon, Munich, Germany).

- Double support (DS) for one leg was defined as the time from its heel strike to toe-off of the opposite leg.
- Step duration (SD) for one leg was defined as the time between its heel strike and the heel strike of the opposite leg.

Symmetry measures for double support and step duration were computed for each phase of the SBT protocol as follows:


$$\text{Symmetr}y_{\left(\mathrm{DS}\right)}=\frac{\text{mean}{\left(\mathrm{DS}\right)}_{\text{fast}}-\text{mean}{\left(\mathrm{DS}\right)}_{\text{slow}}\;}{\text{mean}{\left(\mathrm{DS}\right)}_{\text{fast}}+\text{mean}{\left(\mathrm{DS}\right)}_{\text{slow}}}$$

$$\text{Symmetr}y_{\left(\mathrm{SD}\right)}=\frac{\text{mean}{\left(\mathrm{SD}\right)}_{\text{slow}}-\text{mean}{\left(\mathrm{SD}\right)}_{\text{fast}}\;}{\text{mean}{\left(\mathrm{SD}\right)}_{\text{slow}}+\text{mean}{\left(\mathrm{SD}\right)}_{\text{fast}}}$$

Symmetry at baseline was defined as the mean of the last 10 steps during tied-belt period of baseline, early adaptation (EA) the mean of steps 5 to 10 of the adaptation period, late adaptation (LA) the mean of the last 10 steps of the adaptation phase. Due to the length of the adaptation phase and individual variability in the rate of adaptation, many participants adapted before the end of the adaptation phase. To accurately evaluate the adaptation symmetry, an adaptation plateau (Plat_A) was defined as the point of minimum asymmetry, identified using a 50-step moving average to smooth the data. The same approach was applied to the postadaptation phase, with the postadaptation plateau (Plat_PA) representing the point of minimal asymmetry in the opposite direction. Rates of adaptation and deadaptation were defined as the change in symmetry from EA to LA and from early to late postadaptation divided by the total number of steps of the respective period.

### Magnetic resonance imaging acquisition

Functional and structural brain images were acquired on a 3 T Magnetom Prisma scanner (Siemens Healthcare, Germany). 2D gradient echo planar imaging (EPI) was performed with the following parameters: TE/TR = 30 ms/2 s, 360 volumes in time, acquisition matrix = 230 × 230, slice thickness = 2 mm, 35 slices, FOV = 230 × 230 mm^2^ and total acquisition duration of 12 min. An anatomical T1-weighted image was acquired with the following sequence parameters: MPRAGE 3D T1-weighted sagittal volume, TE/TR/ TI = 3.17 ms/2.4 s/1000 ms, acquisition matrix = 256 × 256 × 176, field of view (FOV) = 256 × 256 mm^2^, 210 Hz/px readout bandwidth, flip angle = 8 degrees and total acquisition duration of 5.42 min. MRI images were evaluated by a neuroradiologist only in the event abnormalities in the images were identified by the experimenter.

### Voxel based morphometry

T1-weighted images were segmented into grey and white matter and cerebrospinal fluid using CAT12 (Computational Anatomy Toolbox, Jena, Germany) toolbox standard pipeline in MATLAB 2020b. The images were normalized to CAT12’s predefined template using Geodesic Shooting (Ashburner and Friston, 2011) and smoothed using a full width at half maximum (FWHM) Gaussian kernel of 8 mm^3^. Total intracranial volume (TIV), grey matter, white matter and cerebrospinal fluid volumes were estimated. Additionally, the volumes of our regions of interests (ROIs), within the motor and locomotor networks including primary motor cortex (M1), cerebellum, supplementary motor area (SMA), premotor area (PMA), left superior parietal lobe (LSPL) and right superior frontal gyrus (RSFG), as defined by the Neuromorphometrics atlas within CAT12^1^ were estimated.

### Resting state-functional connectivity

T1-weighted images and resting state BOLD fMRI images were preprocessed and denoised using CONN functional connectivity toolbox utilizing the default parameters (Nieto-Castanon, 2020). An ROI-to-ROI connectivity matrix was then computed to characterize functional connectivity within key motor and locomotor network regions.

Motor network ROIs included the primary motor cortex (M1), supplementary motor area (SMA), premotor area (PMA), left superior parietal lobe (LSPL), and right superior frontal gyrus (RSFG), defined using the Human Motor Area Template (Mayka et al., 2006) and Automated anatomical labelling atlas (AAL3) (Rolls et al., 2020). Locomotor network ROIs included the pedunculopontine nucleus (PPN) and the cerebellar locomotor region (CLR), defined following Boyne et al. (2018). The PPN was represented by bilateral spherical seeds with 3 mm radius centered at MNI coordinates −6, −27, −15 and 6, −27, −15; the CLR as a bilateral seed with 6 mm radius spheres centered at −8, −42, −26 and 8, −44, −26.

Functional connectivity strength was calculated as Fisher-transformed bivariate correlation coefficients derived from a weighted general linear model, estimating pairwise associations between BOLD signal time series for each seed-target ROI pair (Nieto-Castanon, 2020).

### Study design

Participants first completed questionnaires assessing cognitive reserve, physical activity history, and handedness, followed by cognitive and motor function tests. They then performed the LAT on the split-belt treadmill and the VAT. In most cases, questionnaires and behavioural assessments were conducted in a single session. MRI scans were acquired either on the same day or within 2 weeks before or after the behavioral session.

### Statistical analyses

Correlation analyses were performed to evaluate the relationship between age and participant’s demographic, motor and cognitive abilities. To assess the impact of age on brain structure and function, age was also correlated with regional brain volumes and resting-state functional connectivity (rs-fc) within predefined cortical motor and subcortical locomotor regions.

To evaluate VAT performance, repeated-measures analyses of variances (ANOVA) with Tukey *post hoc* tests were performed on angular deviation errors across task phases [baseline, EA, LA, early postadaptation (EPA), late postadaptation (LPA)]. The influence of age on visuomotor adaptation was assessed by correlating age with error at key VAT phases.

Similarly, for the LAT, repeated-measures ANOVAs with Tukey post hoc comparisons were used to analyze changes in step duration symmetry and double support symmetry across the following phases: baseline, EA, Plat_A, EPA, and Plat_PA. Age-related effects were examined by correlating age with symmetry measures at critical LAT phases. Mauchly’s test was used to assess sphericity; when violated, Greenhouse–Geisser corrections were applied.

To explore the role of brain structure and function in adaptation, volumes and rs-fc of predefined motor and locomotor regions were correlated with VAT and LAT performance measures. Additionally, to assess the contribution of lifestyle and biographical factors, self-reported physical activity (adolescent and recent) was correlated with both adaptive performance and brain measures found to support adaptation.

Spearman’s correlation was used throughout due to the modest sample size and its robustness to outliers and violations of normality. Benjamini-Hochberg corrections (q < 0.050) with multiple comparisons were applied to correct for the volume of the three tissue classes (GM, WM and CSF), volume of 7 regions of the motor and locomotor network selected *a priori*, and 8 predefined ROI-to-ROI pair rs-fcs.

## Results

### Demographic and psychometric data

The 24 participants (13 women) ranged in age from 21 to 80 years (mean = 47.2 ± 18.4). Two-tailed Spearman’s correlations revealed significant associations between age and scores on the MoCA, FAB, FGA, BDI, CRIq, and six-minute walk test (6MWT). No significant correlations were found between age and TUG or Jebsen-Taylor (JT) performance. Additionally, biographical measures of motor reserve, including recent physical activity (SIMPAQ), adolescent physical activity (HistPAQ_e), and recent physical activity history (HistPAQ_r), were not significantly correlated with age. Demographic characteristics and correlation results are summarized in Table 1.

**Table 1:** Demographic, motor and cognitive assessment scores of the participants and their correlation with age.

Measure	Mean [range]	SD	R_s_	*p*
**MoCA**	**28.71 [26–30]**	**1.40**	**−0.51**	**0.011**
**FAB**	**17.33 [15–18]**	**0.87**	**−0.49**	**0.014**
**FGA**	**29.40 [25–30]**	**1.16**	**−0.43**	**0.035**
**BDI**	**3.79 [0–11]**	**2.60**	**0.47**	**0.018**
**CRIq**	**116.17 [90–153]**	**18.77**	**0.84**	**<0.001**
**6MWT (m)**	**634.6 [540.5–808]**	**71.88**	**−0.54**	**0.007**
TUG (s)	9.06 [6.43–11.38]	1.29	0.15	0.498
JT	0.67 [−0.27–2.53]	0.66	−0.17	0.439
SIMPAQ	2.77 [0.5–5.74]	1.49	0.06	0.790
HistPAQ_e (kcal/week)	2,000.62 [222.8–4,490,2]	1,072.0	0.24	0.263
HistPAQ_r (kcal/week)	2,903.99 [349.0–9840.6]	2,524.0	0.07	0.757

6MWT, six-minute-walking-test; BDI, Beck Depression Inventory; CRIq, Cognitive Reserve Index Questionnaire; FAB, Frontal Assessment Battery; FGA, Functional Gait Assessment; HistPAQ_e, history of physical activity scores for adolescent years (14–20); HistPAQ_r, history of physical activity for most recent age; JT, Jebsen-Taylor hand function test; MoCA, Montreal Cognitive Assessment; SIMPAQ, Simple Physical Activity Questionnaire. Significant correlations at *p* < 0.05 are in bold.

### Neuroimaging correlates of age

One participant did not undergo MRI scans and was not included in any neuroimaging analysis. Participants’ total intracranial volume (TIV), grey matter, white matter, cerebrospinal fluid and regional brain volumes and their correlation with age are summarized in Table 2. Age correlated negatively with grey matter volumes throughout the brain and positively with CSF volume without influencing TIV.

**Table 2:** Total intracranial, GM, WM, cerebrospinal fluid and regional brain volumes of the participants and their correlation with age.

Measure	Mean [range]	SD	R_s_	Fisher’s z	95% CI	*P*_FDR_
TIV (cm^3^)	1,532 [1,341–1,860]	125.60	−0.06	−0.064	−0.464 to 0.358	0.773
**GM (%TIV)**	**44.78 [37.7–51.9]**	**3.31**	**−0.84**	**−1.146**	**−0.919 to 0.609**	**0.002**
**CSF (%TIV)**	**20.11 [11.6–28.8]**	**4.59**	**0.72**	**0.927**	**0.453–0.878**	**0.002**
WM (%TIV)	35.12 [30.9–38.6]	2.35	−0.30	−0.306	−0.632 to 0.132	0.169
**CB (%TIV)**	**6.04 [3.93–7.73]**	**0.85**	**−0.54**	**−0.608**	**−0.78 to −0.168**	**0.005**
**M1 right (%TIV)**	**0.71 [0.52–0.80]**	**0.06**	**−0.66**	**−0.824**	**−0.852 to −0.368**	**0.002**
**M1 left (%TIV)**	**0.73 [0.59–0.85]**	**0.06**	**−0.66**	**−0.813**	**−0.849 to −0.358**	**0.002**
**SMA right (%TIV)**	**0.32 [0.24–0.41]**	**0.04**	**−0.70**	**−0.895**	**−0.870 to −0.428**	**0.002**
**SMA left (%TIV)**	**0.33 [0.27–0.42]**	**0.04**	**−0.69**	**−0.855**	**−0.860 to −0.394**	**0.002**
**SPL left (%TIV)**	**0.59 [0.50–0.73]**	**0.06**	**−0.51**	**−0.566**	**−0.764 to −0.127**	**0.007**
**SFG right (%TIV)**	**0.84 [0.74–1.01]**	**0.07**	**−0.53**	**−0.595**	**−0.775 to −0.155**	**0.007**

CB, cerebellum; CSF, cerebrospinal fluid; GM, grey matter; M1, primary motor cortex; SFG, superior frontal gyrus; SMA, supplementary motor area; SPL, superior parietal lobule; TIV, total intracranial volume; WM, white matter. Significant correlations at *p* < 0.05 are in bold.

The strength of rs-fc between the motor and subcortical locomotor network regions of interest (ROIs) for the participants are summarized in Table 3. No significant correlation between strength of rs-fc and age was found.

**Table 3:** Connectivity between brain regions for 23 participants and their correlation with age.

Measure	Mean (R)	SD	R_s_	Fisher’s z	95% CI	*P*_FDR_
PPN-RSFG	0.003	0.11	0.09	0.119	−0.309 - 0.506	0.487
PPN-RCB	−0.01	0.13	−0.21	−0.261	−0.604 - 0.176	0.487
CLR-LM1	0.18	0.17	−0.02	−0.048	−0.451 - 0.372	0.487
LM1-RPMA	0.25	0.18	0.04	0.069	−0.353 - 0.468	0.487
LM1-RM1	0.86	0.30	−0.08	−0.081	−0.477 - 0.343	0.487
LSMA-RPMA	0.86	0.20	0.12	0.163	−0.269 - 0.538	0.487
LSPL-RPMA	0.19	0.16	0.33	0.387	−0.052 - 0.678	0.487
LPMA-LSMA	0.07	0.20	0.01	0.052	−0.368 - 0.454	0.487

CLR, cerebellar locomotor region; LM1, left primary motor cortex; LPMA, left premotor area; LSMA, left supplementary motor area; LSPL; left superior parietal lobe; PPN, pedunculopontine nucleus; RCB, right cerebellum; RM1, right primary motor cortex; RPMA, right premotor area; RSMA, right supplementary motor area; RSFG, right superior frontal gyrus.

### Visuomotor adaptation task

For technical reasons, two participants’ VAT data had to be excluded due to missing data. Thus, a total of 23 participants were included in the analyses of adaptation and post- adaptation phases, respectively. Repeated-measures ANOVA revealed significant differences in angular deviation across VAT phases [*F*(2.313, 48.57) = 129.3, *p* < 0.001; Figure 2C]. Deviation was highest during EA, decreased by LA, and reversed direction during EPA, gradually returning to baseline by LPA. *Post hoc* comparisons confirmed that each phase differed significantly from all others.

Spearman’s two-tailed correlation showed a significant positive association between age and baseline directional error [Rs(22) = 0.43, *p* = 0.038], possibly reflecting age-related differences in computer mouse familiarity. However, no significant correlations were found between age and adaptation measures in the VAT.

Spearman’s two-tailed correlation analysis with age as covariate revealed significant correlations between TIV and measures of the VAT, which are summarized in Table 4. Additionally, degree of error at LA was negatively associated with CSF volume [R_S_(20) = −0.55, *p*_FDR_ = 0.012] and positively associated with WM volume [R_S_(20) = 0.61, *p*_FDR_ = 0.006]. Degree of error at EA was positively associated with WM volume [R_S_(20) = 0.51, *p*_FDR_ = 0.045]. Cerebellar volume was positively associated with degree of deadaptation error [R_S_(19) = 0.64, *p*_FDR_ = 0.014] and rate of deadaptation [R_S_(19) = 0.65, *p*_FDR_ = 0.007]. No other associations between TIV, volume of the tissue classes, regional brain volume and rs-fc with VAT were found.

**Table 4:** Two-tailed Spearman’s correlations of TIV and VAT with age as covariate.

Measure	R_s_	*P*
Baseline	−0.217	0.332
EA	0.111	0.622
LA	−0.080	0.724
Adaptation (°)	−0.087	0.701
Adaptation rate (°/no. of trials)	−0.206	0.371
**EPA**	**0.456**	**0.038**
LPA	0.166	0.461
**After-effects**	**0.548**	**0.012**
**Deadaptation (°)**	**−0.473**	**0.030**
**Deadaptation rate (°/no. of trials)**	**−0.487**	**0.025**

EA, degree of error at early adaptation; EPA, degree of error at early postadaptation; LA, degree of error at late adaptation; LPA, degree of error at late postadaptation; TIV, total intracranial volume. Significant correlations at *p* < 0.05 are in bold.

### Locomotor adaptation task

LAT data of one participant failed to be recorded, hence, 23 participants were included in all analyses involving LAT. Repeated measures ANOVA and post-hoc tests revealed that DS symmetry significantly worsened at the onset of the adaptation phase, improved at the initial adaptation plateau, but worsened again toward the end of adaptation [*F*(2.25, 47.24) = 28.03, *p* < 0.001]. At EPA, DS symmetry immediately improved and then worsened at LPA (Figure 2B). Repeated measures ANOVA also revealed SD symmetry significantly worsened during EA, only slightly improving towards LA, and asymmetry in the opposite direction was observed at postadaptation [*F*(2.706, 62.25) = 97.61, *p* < 0.001, Figure 1C]. Spearman’s two-tailed correlations revealed that DS symmetry and SD symmetry are significantly anticorrelated during the adaptation phase [R_s_(595) = −0.92, *p* < 0.001; Supplementary Figure 1]. No correlation of age and measures of the LAT was found.

**Figure 3:**
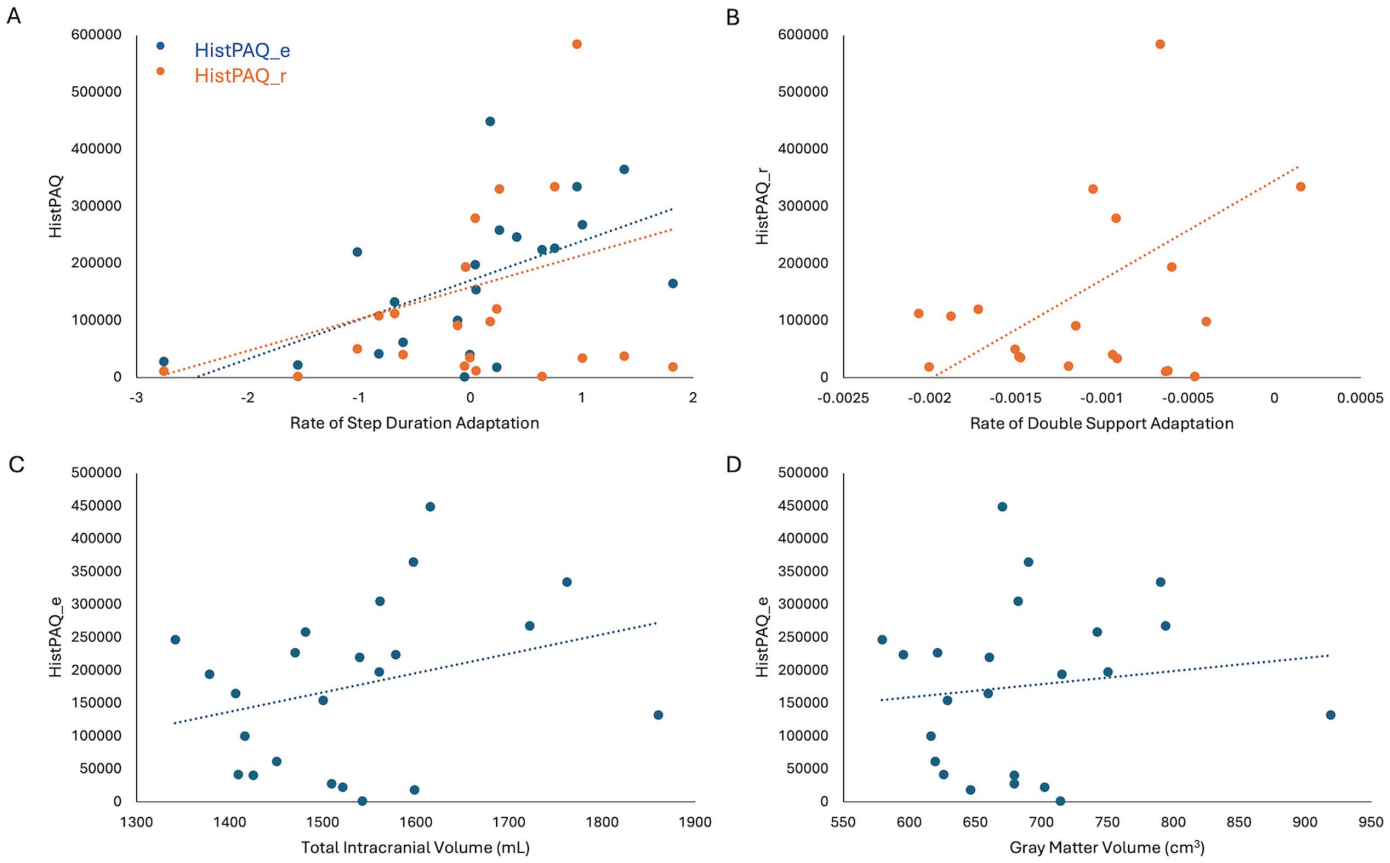
**(A)** Adolescent (HistPAQ_e) and recent (HistPAQ_r) physical activity is positively associated with rate if step duration adaptation. **(B)** Rate of Double Support adaptation is positively associated with recent physical activity. **(C,D)** Adolescent physical activity is positively associated with total intracranial volume **(C)** and absolute gray matter volume **(D)**. HistPAQ_e, history of early physical activity; HistPAQ_r, recent history of physical activity.

Spearman’s two-tailed correlations with age as covariate revealed that total intracranial volume was positively associated with SD and negatively associated with DS symmetry at baseline [SD: R_s_(19) = 0.48, *p* = 0.026; DS: R_s_(19) = −0.44, *p* = 0.046]. Additionally, SD symmetry at LPA was positively associated with total intracranial volume [R_s_(19) = 0.51, *p* = 0.018]. Spearman’s two-tailed correlation analyses further revealed that double support symmetry at late adaptation was positively associated with rs-fc between left M1 and right PMA [R_s_(20) = 0.64, *p*_FDR_ = 0.016], such that stronger connectivity reflected greater asymmetry. No other associations between TIV, volume of the tissue classes, regional brain volume and rs-fc with LAT were found (Figure 3).

### Influence of physical activity on motor adaptation and underlying neural mechanisms

No significant correlations were found between HistPAQ and VAT measures. However, Spearman’s two-tailed correlations revealed that physical activity during adolescence positively correlated with rate of SD adaptation [R_s_ (21) = 0.57, *p* = 0.005] and negatively with DS symmetry at EA [R_s_ (21) = −0.42, *p* = 0.046]. Recent physical activity (HistPAQ_r) was positively associated with the rate of SD [R_s_(21) = 0.60, *p* = 0.003] and DS adaptation [R_s_(21) = 0.48, *p* = 0.026].

To explore neural mechanisms underlying these associations, Spearman’s correlations (controlling for age) were conducted between physical activity and brain structural and functional measures. HistPAQ_e was positively correlated with total intracranial volume (TIV) [R_s_(20) = 0.46, *p* = 0.030] and absolute GMV [R_s_(20) = 0.60, *p* = 0.003]. Most recent physical activity, measured by the SIMPAQ, was negatively correlated with CSF volume [R_s_(20) = −0.58, *p*_FDR_ = 0.015].

## Discussion

This cross-sectional study examined biographical, behavioral, and neuroimaging measures to capture individual motor adaptive capacity. We first investigated the impact of age on motor behavior, brain structure, and brain function. As expected, brain volume declined with age, and older adults walked shorter distances in 6 min. However, both visuomotor and locomotor adaptation were preserved across the lifespan. Adaptation ability was linked to brain volume and function, particularly in motor and locomotor network regions. Importantly, brain structure in these regions were associated with both adolescent and current physical activity levels. These findings suggest that lifestyle-modifiable differences in brain structure may help preserve motor adaptation despite general structural decline. Overall, visuomotor and locomotor adaptation may serve as surrogate markers of a motor reserve, a capacity shaped by physical activity that supports structural and functional integrity of motor networks, promoting resilience to aging and disease.

### Motor adaptation is largely preserved across the lifespan

#### Visuomotor adaptation

Declines in the rate and magnitude of visuomotor adaptation in older adults have been reported repeatedly (Heuer and Hegele, 2014; Buch et al., 2003; Li et al., 2021; Vachon et al., 2020). However, several studies show that when no explicit instructions are provided, older adults adapt at rates and magnitudes comparable to younger adults. This suggests that while older adults may be less efficient at using explicit strategies, implicit adaptation via visual feedback remains intact (Buch et al., 2003; Vachon et al., 2020; Li et al., 2021). Since our participants received no explicit instructions, it is unsurprising that older adults in our sample adapted as well as younger adults. Moreover, the smaller perturbation used in our study (30° vs. 75° in Heuer and Hegele, 2014) may have reduced sensitivity to age-related differences in directional error, implying that such differences may emerge only under larger perturbations or with explicit instruction.

#### Locomotor adaptation

Consistent with previous evidence, we found that the *magnitude* of locomotor adaptation does not decline with age (Ducharme et al., 2019; Malone and Bastian, 2016; Roemmich et al., 2014; Sato and Choi, 2022; Sombric et al., 2017; Vervoort et al., 2019). While some studies have reported a slower adaptation rate in older adults, our results did not support this (Bruijn et al., 2012; Sombric et al., 2017). These previous studies, however, did not tailor treadmill belt speeds to individual walking speeds, making it difficult to separate age effects from general performance differences. Furthermore, they also included more participants over 70 years old (e.g., Bruijn et al., mean age 73.1, *N* = 12; Sombric et al., 2017, mean age 77.2, *N* = 8). Indeed, Bruijn et al. (2012) found that the age–adaptation rate correlation disappeared when the youngest and oldest participants were excluded, suggesting that differences are driven largely by individuals at the extremes of the age spectrum. Moreover, significant reductions in adaptation rate are typically observed only beyond age 70 (Sato and Choi, 2022). Thus, our limited sample of older adults may have reduced sensitivity to such effects. While older adults appear to adapt at rates similar to younger adults, they may show reduced retention or consolidation (Malone and Bastian, 2016; Sombric et al., 2017), possibly reflecting compensatory strategies to maintain gait stability.

Another factor supporting preserved adaptation in our sample could be their high physical activity levels. Our participants averaged 156 min of daily activity, far exceeding the U. S. average of 10–30 min (Troiano et al., 2008). Given our finding that physical activity levels are linked to adaptation rate, this elevated activity likely contributed to their maintained adaptive capacity. Under individually adjusted testing conditions, locomotor adaptation appears largely preserved in physically active older adults.

### Physical activity has little impact on visuomotor adaptation

In this cohort of generally physically active adults, neither age nor levels of physical activity, whether recent or during adolescence, appeared to significantly influence visuomotor adaptation. This aligns with previous findings showing no substantial effect of exercise on visuomotor rotation adaptation (Ferrer-Uris et al., 2017; James and Wang, 2023).

### The cerebellum—a mainstay for visuomotor adaptive capacity

To investigate the neural basis of preserved visuomotor adaptation in aging, independent of exercise, we used structural and functional neuroimaging.

As expected, better performance on the VAT, particularly faster and greater deadaptation, was positively associated with gray matter volume and structural integrity of cerebellum. This adds to the growing evidence from structural and functional imaging that that visuomotor adaptation relies on the cerebellum (Cassady et al., 2018; Tomassini et al., 2011; Wolpe et al., 2020). Indeed, cerebellar-based motor learning appears to remain intact in aging (Wolpe et al., 2020) and in diseases such as Multiple Sclerosis (Nguemeni et al., 2020) and may even compensate when primary motor areas are impaired (Bonni et al., 2020). This suggests that cerebellar learning mechanisms could be leveraged therapeutically, either to compensate for existing deficits or to bolster motor function in anticipation of age- or disease-related decline. Brain stimulation methods, both invasive, by deep brain stimulation, and non-invasive, by transcranial direct current stimulation targeting cerebellum has shown promise in the treatment of stroke and movement disorders (Fox-Hesling et al., 2024; Baker et al., 2023; Tai and Tseng, 2022).

Overall, the cerebellum likely plays a central role in motor adaptability. Structural and resting-state functional properties of the cerebellum, combined with behavioral measures like visuomotor adaptation, may serve as proxies for an individual’s motor reserve. However, determining causality, whether superior performance results from stronger brain structure and connectivity or vice versa, will require longitudinal research.

### Interhemispheric motor connectivity may enhance locomotor adaptability

Unlike the VAT, we found no strong association between the structure or function of the cerebellar locomotor network and LAT. The limited variability in locomotor adaptation ability and network structural integrity may have reduced our sensitivity to detect such links. However, weaker connectivity between left M1 and right PMA was associated with better DS symmetry in late adaptation. This aligns with evidence that unilateral movements benefit from interhemispheric inhibition between motor cortices (Kajal et al., 2017; Morishita et al., 2012). Enhanced interhemispheric inhibition may enable more efficient, specialized motor control, thereby supporting adaptive performance.

### Physical activity may promote locomotor adaptive capacity by brain capacity maintenance

A key finding in our study is that physically active adults, regardless of age, demonstrate superior locomotor adaptation rates on the split belt treadmill compared to their less active peers. This highlights the critical role of physical activity in preserving motor adaptive capacity despite age-related motor system decline. Given that successful motor adaptation has been linked to differential recruitment of neural networks, we also used neuroimaging to more directly explore the underlying neural mechanisms.

We found that total intracranial volume and absolute gray matter volume were positively correlated with adolescent, but not recent, physical activity levels. Consistent with studies showing that aerobic exercise enhances neuroplasticity (Mattson, 2015; Raichlen and Alexander, 2017), this suggests that physical activity during adolescence may enhance brain volume (Estevez-Lopez et al., 2023; Lenroot and Giedd, 2006). Total intracranial volume, in turn, positively correlated with symmetry of step duration during late postadaptation, indicating that individuals with greater brain volume, potentially reflecting greater neural resources, may better compensate under motor challenges.

These findings support the adaptive capacity model, which links physical activity to sustained brain capacity and motor adaptability, while inactivity leads to diminished reserve (Raichlen and Alexander, 2017). Early-life physical activity may boost brain tissue volume and provide resilience against age-related atrophy (Colcombe et al., 2006; Jeon et al., 2024). Long-term exercise has been linked to increased superior frontal cortex volume and reduced brain atrophy, reinforcing its protective role (Arnardottir et al., 2016; Bugg and Head, 2011). Thus, individuals with lower adaptive capacity might benefit from regular physical activity to mitigate age- and disease-related decline. While these associations are promising, establishing causality between physical activity and brain structure will require longitudinal studies to disentangle directionality.

### Limitations

The primary limitation is the small sample size of neurologically healthy participants with moderate variability of physical activity, which may have limited the detection of significant correlations. However, because motor adaptation was largely preserved in our sample, it is essential to examine whether similar neural and behavioral associations emerge in populations with reduced motor reserve due to aging or disease. While our methods do not allow for causal conclusions, the findings support a protective role of physical activity in neurological health and highlight the need for longitudinal research. In order to control for computer mouse familiarity in a cohort of a such a wide age range, we recommend future studies consider adding this as a covariate or incorporating a pre-acclimatization.

The use of the BDI represents another limitation as it may overestimate depression in older adults due to the presence of somatic items (Trentini et al., 2005). However, it remains a valid and reliable tool for adults up to age 80, provided they do not have sleep disturbances (Segal et al., 2008; Jefferson et al., 2001; Olin et al., 1992). Additional limitations include disruptions caused by the COVID-19 pandemic and reliance on self-reported physical activity data, the latter of which could be mitigated in future studies through the use of wearable activity monitors.

## Conclusion

Motor adaptability is maintained by the functional integrity of the motor and locomotor network despite declines in brain structure due to aging. Lifelong physical activity may help build a structural reserve, enhancing the system’s capacity to handle challenges. Measures of current motor adaptation, along with MRI-based functional connectivity, are promising candidates of individual motor reserve (mR). While these associations are promising, establishing causality between physical activity and brain structure will require longitudinal studies to disentangle directionality. However, larger longitudinal studies are needed to establish causal relationships and identify additional contributing factors. Crucially, future studies should include clinical populations and ultimately apply interventional approaches. A better understanding of mR will improve prognostic accuracy and support personalized prevention and treatment of age- and disease-related motor decline by providing objective measures of motor health.

## Data Availability

The raw data supporting the conclusions of this article will be made available by the authors, without undue reservation.

## Footnotes

^1^ http://Neuromorphometrics.com/

## Supplementary material

Supplementary Figure 1 Step duration and double support symmetries are linearly and inversely correlated.
