# Supplementary figures and images for: Unlocking motor reserve: behavioral and neuroimaging correlates of locomotor adaptability from youth to old age
Source: Front Syst Neurosci. 2025 Nov 10;19:1686509. doi: 10.3389/fnsys.2025.1686509 (PMC12640994; doi:10.3389/fnsys.2025.1686509)

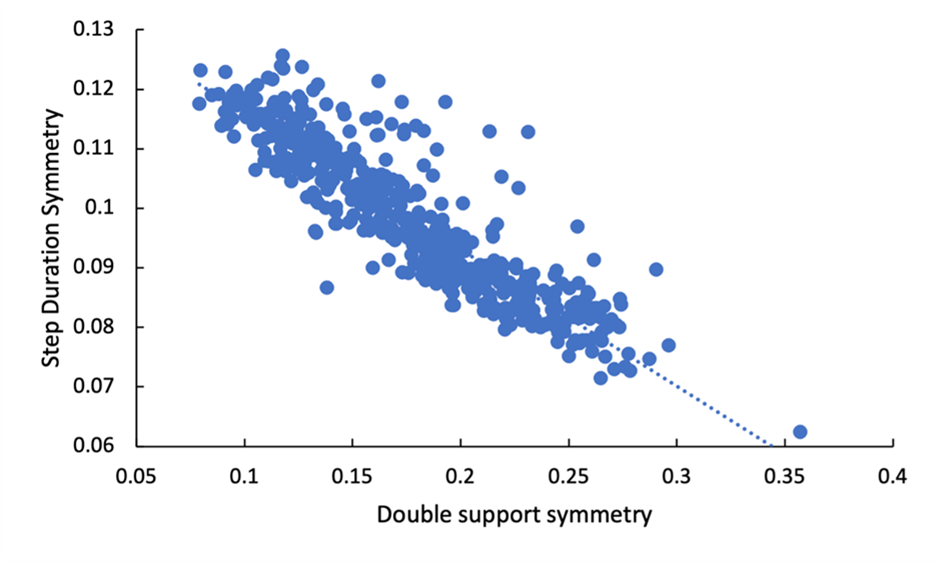

Supplement: Supplementary Figure 1 — Step duration and double support symmetries are linearly and inversely correlated. [file Image_1.tif]
