# Supplementary material for: Unlocking motor reserve: behavioral and neuroimaging correlates of locomotor adaptability from youth to old age
Source: Front Syst Neurosci. 2025 Nov 10;19:1686509. doi: 10.3389/fnsys.2025.1686509 (PMC12640994; doi:10.3389/fnsys.2025.1686509)
Supplement: Supplementary file 2 [file Table_1.docx]

*Supplementary Table 1.*

Details on participant’s history of education and physical activity during earlier years.

| Measure | Mean [range] | SD |
| --- | --- | --- |
| Years of education | 17.63 [11.5-23] | 2.83 |
| Highest level of occupation | 2.79 [0-5] | 1.47 |
| Distance walked to primary school (km) | 1,88 [0.5-5] | 1.43 |
| No. of sports performed during adolescence | 4.33 [1-7] | 1.71 |
| Duration of PA during adolescence (h/week) | 3.92 [0.35-7.64] | 1.93 |

Abbreviations: PA, physical activity.

Elaborations: Level of occupation as defined by CRIq (Nucci, Mapelli, and Mondini 2012). Level 1: unskilled labour; Level 2: craftsperson or skilled labourer; Level 3: business owner, white-collar employee etc.; Level 4: managing director of a small company, qualified freelance professional, high level serviceperson, etc.; Level 5: highly specialized intellectual or scientific professional, etc..; university students without working experience being assigned Level 0. Distance walked to primary school according to HistPAQ (Kriska et al. 1988) in approximations (calculation) of <1 (0.5) km, 1-2 (1.5) km, 3-5 (4) km.

*Supplementary Table 2.*

Demographic, motor, and cognitive assessment scores of the participants by age groups.

| Measure | Age group |  | Mean [range] | SD |
| --- | --- | --- | --- | --- |
| **MoCA** | **All (n=24)** |  | **28.71 [26-30]** | **1.40** |
|  | 18-39 (n=9) |  | 29.33 [29-30] | 0.5 |
|  | 40-59 (n=8) |  | 29 [27-30] | 1.2 |
|  | ≥ 60 (n=7) |  | 27.57 [26-30] | 1.81 |
| **FAB** | **All** |  | **17.33 [15-18]** | **0.87** |
|  | 18-39 |  | 17.56 [16-18] | 0.73 |
|  | 40-59 |  | 17.63 [17-18] | 0.52 |
|  | ≥ 60 |  | 16.71 [15-18] | 1.11 |
| **FGA** | **All** |  | **29.40 [25-30]** | **1.16** |
|  | 18-39 |  | 29.78 [29-30] | 0.44 |
|  | 40-59 |  | 29.75 [29-30] | 0.46 |
|  | ≥ 60 |  | 28.43 [25-29] | 1.81 |
| **BDI** | **All** |  | **3.79 [0-11]** | **2.60** |
|  | 18-39 |  | 2.89 [0-5] | 1.76 |
|  | 40-59 |  | 4 [0-11] | 3.74 |
|  | ≥ 60 |  | 4.71 [1-6] | 1.8 |
| **CRIq** | **All** |  | **116.17 [90-153]** | **18.77** |
|  | 18-39 |  | 95.56 [90-105] | 5.57 |
|  | 40-59 |  | 125.88 [111-137] | 9.98 |
|  | ≥ 60 |  | 131.57 [112-153] | 12.35 |
| **6MWT (m)** | **All** |  | **634.6 [540.5-808]** | **71.88** |
|  | 18-39 |  |  |  |
|  | 40-59 |  |  |  |
|  | ≥ 60 |  |  |  |
| TUG (s) | **All** |  | **9.06 [6.43-11.38]** | **1.29** |
|  | 18-39 |  |  |  |
|  | 40-59 |  |  |  |
|  | ≥ 60 |  |  |  |
| JT | **All** |  | **0.67 [-0.27-2.53]** | **0.66** |
|  | 18-39 |  |  |  |
|  | 40-59 |  |  |  |
|  | ≥ 60 |  |  |  |
| SIMPAQ | **All** |  | **2.77 [0.5-5.74]** | **1.49** |
|  | 18-39 |  | 2,53 [0.5-5.5] | 1.48 |
|  | 40-59 |  | 3.04 [1-5.74] | 1.63 |
|  | ≥ 60 |  | 2.77 [1.14-5] | 1.53 |
| HistPAQ_e (kcal/week) | **All** |  | **2000.62 [222.75-4490.15]** | **1072.0** |
|  | 18-39 |  | 1573.55 [222.75-3648.15] | 1130.26 |
|  | 40-59 |  | 1841.39 [416.19-4490.15] | 1427.28 |
|  | ≥ 60 |  | 2408.65 [1805.1-3053.53] | 406.96 |
| HistPAQ_r (kcal/week) | **All** |  | **2903.99 [349.0-9840.6]** | **2524.0** |
|  | 18-39 |  | 1985.22 [348.99-3722.4] | 1104.37 |
|  | 40-59 |  | 3286.88 [404.53-9840.6] | 3324.38 |
|  | ≥ 60 |  | 3315.52 [1096.1-9711.33] | 2958.67 |

Abbreviations: 6MWT, six-minute-walking-test; BDI, Beck Depression Inventory; CRIq, Cognitive Reserve Index Questionnaire; FAB, Frontal Assessment Battery; FGA, Functional Gait Assessment; HistPAQ_e, history of physical activity scores for adolescent years (14-20); HistPAQ_r, history of physical activity for most recent age; JT, Jebsen-Taylor hand function test; MoCA, Montreal Cognitive Assessment; SIMPAQ, Simple Physical Activity Questionnaire.
